# Effectiveness of neuromuscular electrical stimulation for wrist rehabilitation after acute ischemic stroke: Erratum

**DOI:** 10.1097/MD.0000000000013398

**Published:** 2018-11-16

In the article, “Effectiveness of neuromuscular electrical stimulation for wrist rehabilitation after acute ischemic stroke”,^[1]^ which appeared in Volume 97, Issue 38 of *Medicine*, the corresponding author information should be “Bai-ya Fan, Department of Neurology, The People's Hospital of Yan’an, No. 57 Qilipu St, Baota Qu, Yan’an, 716000, China (e-mails: fbya123@163.com).”
